# Data-Driven Information Extraction from Chinese Electronic Medical Records

**DOI:** 10.1371/journal.pone.0136270

**Published:** 2015-08-21

**Authors:** Dong Xu, Meizhuo Zhang, Tianwan Zhao, Chen Ge, Weiguo Gao, Jia Wei, Kenny Q. Zhu

**Affiliations:** 1 Department of Computer Science & Engineering, Shanghai Jiao Tong University, 800 Dongchuan Road, Shanghai 200240, P.R. China; 2 R & D information China, AstraZeneca, 199 Liangjing Road, Pudong, Shanghai, 201203, China; Jiangnan University, CHINA

## Abstract

**Objective:**

This study aims to propose a data-driven framework that takes unstructured free text narratives in Chinese Electronic Medical Records (EMRs) as input and converts them into structured time-event-description triples, where the description is either an elaboration or an outcome of the medical event.

**Materials and Methods:**

Our framework uses a hybrid approach. It consists of constructing cross-domain core medical lexica, an unsupervised, iterative algorithm to accrue more accurate terms into the lexica, rules to address Chinese writing conventions and temporal descriptors, and a Support Vector Machine (SVM) algorithm that innovatively utilizes Normalized Google Distance (NGD) to estimate the correlation between medical events and their descriptions.

**Results:**

The effectiveness of the framework was demonstrated with a dataset of 24,817 de-identified Chinese EMRs. The cross-domain medical lexica were capable of recognizing terms with an F1-score of 0.896. 98.5% of recorded medical events were linked to temporal descriptors. The NGD SVM description-event matching achieved an F1-score of 0.874. The end-to-end time-event-description extraction of our framework achieved an F1-score of 0.846.

**Discussion:**

In terms of named entity recognition, the proposed framework outperforms state-of-the-art supervised learning algorithms (F1-score: 0.896 vs. 0.886). In event-description association, the NGD SVM is superior to SVM using only local context and semantic features (F1-score: 0.874 vs. 0.838).

**Conclusions:**

The framework is data-driven, weakly supervised, and robust against the variations and noises that tend to occur in a large corpus. It addresses Chinese medical writing conventions and variations in writing styles through patterns used for discovering new terms and rules for updating the lexica.

## Introduction

**Fig 1:**
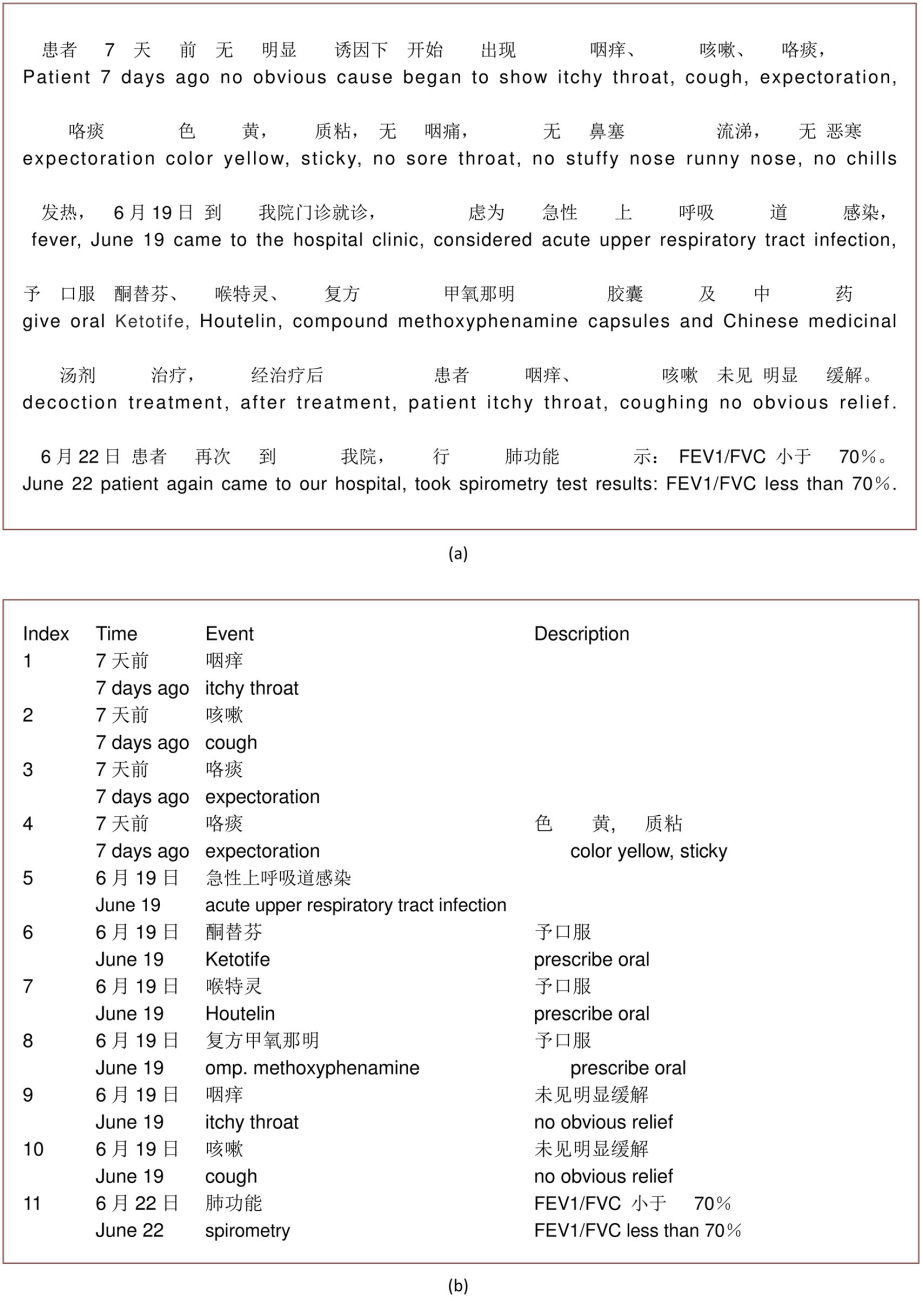
An example of Chinese EMR and structured records extracted from this EMR. (a)a paragraph from an HPI in Chinese with inline literal translation in English. (b)time-event-description triples in Chinese with inline literal translation in English.

Electronic medical records (EMRs) contain valuable information on diseases, examination findings, detailed treatments and outcomes. Extracting such information helps medical professionals understand the natural course of disease, determine the effectiveness of treatments and uncover unmet medical needs. Fig 1a shows a section of an EMR with a narrative of the history of present illness in Chinese. In this paper, we propose an automatic framework that converts such a free text narrative into structured records as shown in Fig 1b. Each output record contains a medical event (potentially a disease, a drug, a symptom, a procedure or a clinical test), the time of the event and an optional description, which elaborates supplementary information on the event. For example, in the fourth record in Fig 1b, the description shows the type of the sputum and its physical properties. Also, this description can be the outcome of a clinical test, such as in the tenth record in Fig 1b, where “FEV1/FVC less than 70%” is the measured result of “spirometry”. Information contained in these structured records is easily accessible and very useful from a real world evidence point of view. Such records, for example, can help physicians assess whether “Ketotife”, “Houtelin”, and “compound methoxyphenamine” are effective or not in relieving the patient’s symptoms.

**Fig 2:**
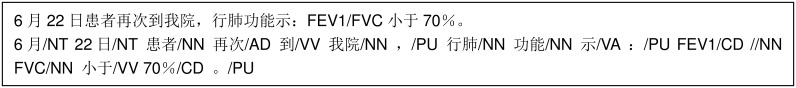
An example of word segmentation and part-of-speech tagging in Chinese.

Automatic data extraction from unstructured medical text like Fig 1a is an intricate task. EMR narratives are often recorded while the physician interviews the patient and are hence, very concise and stylized. Although there are practical standards for medical records writing, significant variations and sometimes errors exist in these texts, attributable to the writing habits and prior knowledge of the authors. For example, while “上呼吸道感染” (upper respiratory tract infection) is the official, full name of the disease, some Chinese physicians write “上感” (literally “upper infection”) as an informal abbreviation. Furthermore, to effectively extract structured records from Chinese EMRs, one must address the additional challenges posed by the high density of the Chinese language compared with English [1]. Specifically, Chinese EMRs written by doctors practicing Traditional Chinese Medicine (TCM), can appear very similar to ancient Chinese texts. This makes the Chinese Natural Language Processing (NLP) models trained from standard, modern text such as news articles or common literature no longer valid or effective. Fig 2 shows the result of word segmentation and part-of-speech tagging of the last sentence in Fig 1a by the Stanford NLP tools [2,3]. Here, “行” (take) and “肺”(lung) are incorrectly grouped together, which make it impossible to recognize the term “肺功能” (lung function). Annotating a large quantity of Chinese EMRs to train new models is an option but is extremely costly for medical texts because significant domain knowledge is required.

Herein, we focus on two main tasks. The first one is medical event recognition in EMR narratives, which can be categorized as a Named Entity Recognition (NER) problem. As one of the major tasks in NLP, existing methods for NER generally fall into four classes, namely pattern-based [4, 5, 6, 7], knowledge-based [8], supervised learning-based [9,10] and hybrid approaches [11]. A lot of work has been proposed to apply these approaches to medical events recognition in EMRs. Sirohi *et al* [12] and Sohn *et al* [13] developed dictionary-based systems to extract drug information from clinical texts. Voorham *et al* [14] proposed a pattern-based approach to extract numeric clinical measurements related to diabetes care from free text in EMRs. However, because of synonyms and morphologic variants to medical terms, those methods are mostly simplistic and suffer from low coverage and accuracy [15]. In addition, the construction of patterns is corpus-dependent and always requires domain knowledge, which makes it difficult to scale. On the other hand, many supervised learning approaches have been used in medication information extraction problems recently [16, 17, 18]. Tang *et al* [19] implemented structural support vector machine (SVM) based on features such as bag-of-word, part-of-speech and discourse information to recognize clinical entities. Though these approaches have been reported to achieve high accuracy, a large annotated corpus is required. The availability of such a corpus in Chinese is limited. As EMRs in Chinese become increasingly available, a number of systems were proposed to extract clinical information from Chinese EMRs [20, 21, 22]. Liu *et al* [23] tried to discover knowledge related to TCM from clinical data of daily clinical operations. Lei *et al* [24] systematically investigated the performance of supervised learning such as conditional random fields (CRF), Maximum Entropy (ME), SVM and structural SVM in medical entity recognition in Chinese EMRs. Representatives of the state-of-the-art results will be compared with our work in this paper.

The second task of this effort is to establish an association between medical events and descriptions. Jiang *et al* [17] developed a rule-based system to associate descriptions with clinical entities. Aramaki *et al* [25] proposed a model combining patterns and an SVM classifier to identify the relationship between drugs and their effects. Patrick and Li [26] implemented an SVM classifier, consisting of local context features and semantic features, to identify the relationship between medical terms. However, the descriptions are always sparse. For example, there are many possible descriptions for “咳痰” (expectoration), such as “色黄,质粘” (literally “color yellow, sticky”) or “白色泡沫” (white foam) and so on. In this situation, only considering local or semantic features is not enough. The Normalized Google Distance (NGD) proposed by Cilibrasi *et al* [27] provides us with a suitable tool to address this issue. Based on the World Wide Web, we can easily estimate the relatedness of medical events and descriptions. Besides, temporal information is important for many medical applications. A number of studies [28,29,30] have addressed various topics of temporal representation and reasoning with medical data. However, to the best of our knowledge there’s little research on the extraction of temporal information from Chinese EMRs.

In summary, we propose a novel, data-driven framework to efficiently extract medical information from Chinese EMR narratives. The framework aims to convert the unstructured free text in Fig 1a into structured records shown in Fig 1b. The proposed framework depends minimally on common NLP tools except for their basic lexical analysis capabilities. It can be considered a hybrid approach, which combines the core lexica of medical terms, an iterative bootstrapping algorithm to accrue more accurate terms into the lexica and an SVM algorithm to compute correlations between terms and descriptions. The methods and algorithms are data-driven, weakly supervised, and robust against variations and noises that are often present in a large corpus.

## Materials and Methods

### Electronic medical records

A real world dataset of 24,817 de-identified EMRs from hospitalized infection patients were collected from the Guangdong Provincial Hospital of Traditional Chinese Medicine (GPHTCM) in Guangdong, China. The study was approved by the Ethics Committee of GPHTCM.

The extraction was performed on the History of Present Illness (HPIs) for demonstration purposes. The targets of extraction can be extended to many other free text sections in the Chinese EMRs, including Chief Complaint (CC), Past Medical History (PMH) and Treatment Records (TR). The resulting structured records contain dated events with an optional description.

The 24,817 HPIs were divided randomly into three parts, a training set containing 24,317 records, a hold-out set of 400 records (GD-400) and a tuning set of 100 records (GD-100). The framework was trained on the training set. Parameter tuning was done on GD-100. All testing and comparisons were done on GD-400. GD-400 and GD-100 were labeled with temporal terms, medical terms and the associated descriptions by two human judges. Where there was disagreement between the two, a third judge was brought in to resolve the difference by selecting and confirming one of the two different annotations. In fact, the two judges agreed on about 95% of the labels. GD-400 contains 9019 medical terms, 1384 temporal terms and 6112 (time-event) tuples. 2262 descriptions are associated with a medical event. GD-100 contains 331 disease terms and 166 drug terms. Other terms in GD-100 were not labeled, as they were not used in parameter turning.

### Extraction workflow

**Fig 3:**
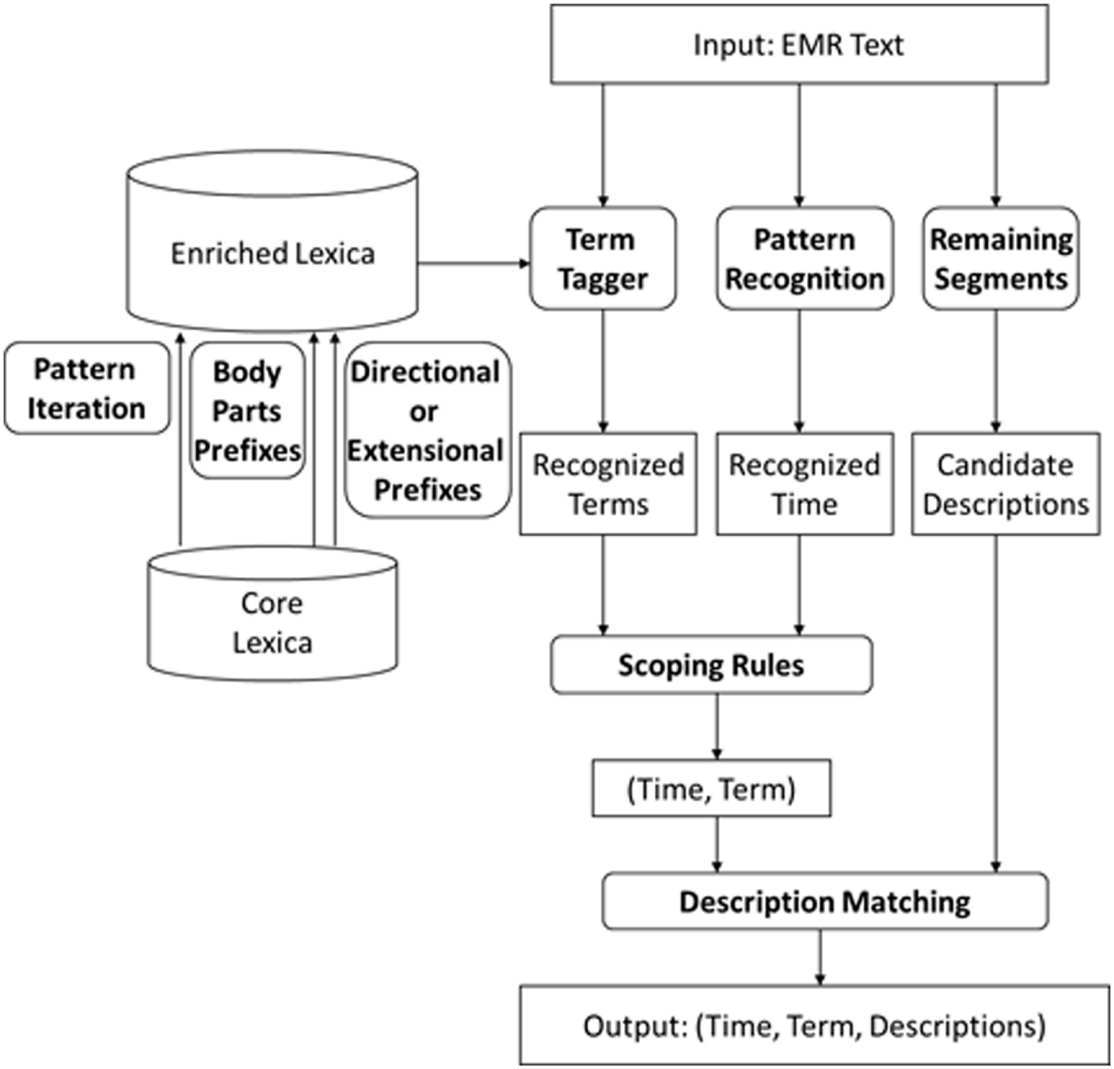
Chinese EMR information extraction system workflow.

To accomplish our goal, we first constructed a comprehensive cross-domain medical dictionary by enriching a collection of core Chinese medical term lexica using pattern iterations, dynamic terms and directional or extensional prefix updates. Then the cross-domain dictionary was used to recognize clinical named entities in the Chinese EMRs. Finally, a statistical model was learned to infer correlations between the events and auxiliary information. This workflow is detailed in Fig 3.

### Cross-domain medical dictionary

Due to the complexity of EMRs compared to natural language, off-the-shelf Chinese NLP parsers can’t be directly applied to help extract candidate terms. Deciphering such texts needs to be driven by term recognition instead. On the other hand, Chinese EMRs contain medical named entities with variations in expression. For example, diseases, drugs and procedures can be referred to by both their official names and abbreviations. Existing medical lexica are insufficient for recognizing all meaningful terms in an EMR. Therefore our first step is to create a large-scale comprehensive Chinese medical dictionary, which includes six types of terms: diseases, symptoms, drugs, body parts, procedures, and clinical tests.

### Resources included in the core lexica

We began by sourcing reliable information both online and offline. The official lexica included were Systematized Nomenclature of Medicine-Clinical Terms (SNOMED-CT) in Chinese [31] and Chinese Pharmacopoeia [32]. The open access medical websites and encyclopedias included were Wanfang Med Online [33], Baidu Baike [34] and Hudong Baike [35]. Also included was the dictionary from the Sogou Chinese Input Method [36], a search engine based input software. The paper-based Chinese Pharmacopoeia was scanned and processed using Optical Character Reading (Abbyy FineReader v10). All other resources were available in an electronic format. The “unofficial” resources contain variations of medical terms that are not of the official form but may appear in the EMRs. For example, some physicians prefer to use “diabetes mellitus”, which is not found in official resources, instead of “noninsulin-dependent diabetes mellitus”. Similarly, the same drug can be referred to by its brand name and generic name. Including them in the lexica improves the recall and accuracy of downstream analyses.

### Enrichment of core lexica by pattern iteration

To further improve recall, a data-driven approach was developed to enrich the core lexica. We used an unsupervised iterative process to bootstrap the existing lexica based on learned patterns. Such pattern iterative bootstrapping methods have been used in the construction of well-known open-domain knowledge bases such as Probase [37], TextRunner [38], NELL [39] and Know-It-All [40].

The algorithm started with the core lexica. In each iteration, it first scanned through the corpus of the EMR and extracted terms in the current lexica. It then tried to discover high quality patterns. In order to generate candidate patterns, we considered up to 3 words (including punctuation marks) to the left and the right of the extracted terms. For example, in the sequence “, 给予头孢呋抗感染” (literal translation: “, prescribe cefuroxime for anti-infection”), “cefuroxime” was recognized as a drug. Nine candidate patterns were then generated as follows:

予<DRUG>抗
予<DRUG>抗感
予<DRUG>抗感染
给予<DRUG>抗
给予<DRUG>抗感
给予<DRUG>抗感染
,给予<DRUG>抗
,给予<DRUG>抗感
,给予<DRUG>抗感染

A large number of candidate patterns were generated following the logic above. Ideally, we would like to keep the patterns that can extract as many as possible previously unknown terms from the corpus. On the other hand, such patterns are more likely to introduce noisy terms into the lexica, resulting in more low quality patterns being harvested as valid patterns. The enriched lexica would then be laden with errors. To avoid this problem, we defined two parameters as follows to evaluate the candidate pattern’s ability to extract terms from the training corpus as well as the precision of terms extracted by this pattern:

Pattern Selectivity(PS) = Number of unique correct terms extracted by P

$$\text{Pattern Precision(PP) }=\;\frac{\text{Number of unique correct terms extracted by P}}{\text{Number of unique terms extracted by P}}$$

Here, a correct term referred to a term that was either included by the original core lexica or was already added into the core lexica by the previous iterations. In order to ensure the quality of patterns, we used thresholds to filter out the unreliable patterns, namely Pattern Selectivity Threshold (PST) and Pattern Precision Threshold (PPT). High quality (i.e., PS > PST and PP > PPT) patterns were kept while the others were discarded.

In the second phase of the iteration, we used the newly discovered patterns to extract new terms (T), which were also scored by the number of patterns they occurred in, namely

Terms Frequency(TF) = Number of Patterns T occurs in

A Term Frequency Threshold (TFT) was set in place to control their quality. Only reliable terms (i.e., TF > TFT) were added to the lexica. The iteration continues until no new terms are extracted.

The thresholds for the iterative algorithm were tuned on GD-100. Empirically, 1 was a reasonable value for TFT. That means if a term can be extracted by more than one pattern, this term is trustworthy. For PST and PPT, a grid-search was performed to discover the optimum parameters. All combinations of PST from 3 to 10 with step length 1, and PPT from 0.3 to 0.9 with step size 0.05 were generated. The configuration that produced the best F-1 score was chosen.

To assess the effect of lexicon enrichment, we implemented the conditional random field (CRF) method for concept recognition proposed by Lei *et al* [24], one of the most recent studies on medical term recognition from Chinese EMRs, as a baseline comparison. We used the bag-of-characters feature for CRF, which reportedly performed among the best. GD-400 was used as the dataset. We used a 5-fold cross validation to evaluate the CRF method. The significance level of the difference in F1-scores was assessed using the Macro t-test, which compares two systems based on the F1-scores of individual categories [41]. A one-sided p-value that is smaller than 0.05 was considered statistically significant. The results are presented in the Results and Discussion section.

### Dynamic term assembly using body part prefixes

**Fig 4:**
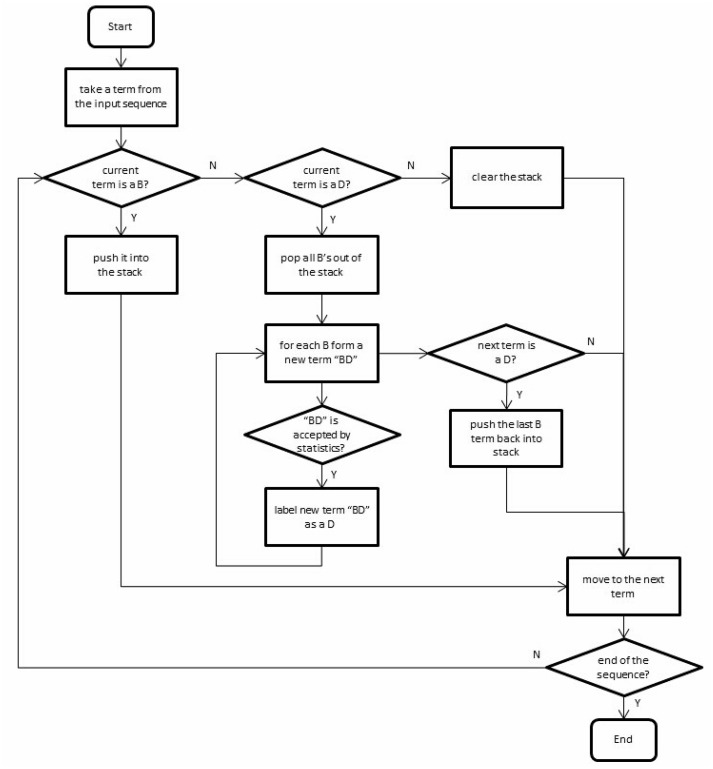
Dynamic term assembly using body part prefixes.

The previous step focused on identifying “stand-alone” terms. The dynamic term assembly step aimed to recognize compound terms that were not readily available in the core lexica, but consisted of multiple stand-alone terms from the core lexica. For example, “oral mucosa bleeding” is a compound term of the symptom category. It is not covered by the core symptom lexicon. However, its components, namely “oral”, “mucosa” and “bleeding”, are each covered by a core lexicon. We have noticed that body parts often act as common prefixes for these compound terms. There are two general cases to be addressed: B_1_, …, B_n_, D and B,D_1_,…, D_n_, where B stands for a body part term and D stands for a disease/symptom/procedure/clinical test term. The first case may be parsed into B_1_D, …, B_n_D, whereas the second case may be parsed into BD_1_, …, BD_n_, according to the Chinese language convention. To avoid mistakes such as parsing “abdominal pain, vomiting” as “abdominal pain, abdominal vomiting”, a data-driven method was implemented to determine the scope of a given prefix in terms of the value of *n*. The co-occurrences of all B terms with all D terms were calculated from the EMR text training data to obtain the probability of a B term followed by a D term. A non-adjacent B-D pair was accepted as a new term if the probability of the two terms occurring directly adjacent to each other was higher than a certain threshold. This process can be thought of as establishing a statistical association between body part terms and other terms. The dynamic parsing of the two types is detailed in Fig 4.

### Directional or extensional prefix update

In many cases, medical terms start with directional or extensional words, such as 左侧输尿管结石 (literal translation—“left ureteral stones”), 右下肺感染 (literal translation “right lung infection”) and 双肾结石 (literal translation “double kidney stones”). However, the core lexica only contain “ureteral stones”, “lung infection” and “kidney stones”. To overcome this limitation, we implemented a three-word look-back. If the words leading to a known medical term were directional words or extensional words they were assembled into a new medical term tagged by the category of the original medical term.

### Extraction of structured records from EMR texts

Extraction was done in two steps. In step 1, we deployed an event tagging algorithm and a set of temporal term recognition rules to recognize medical terms and temporal terms from the text. The remaining segments of the text were considered candidate descriptions of the medical terms. In step 2, each medical term that signifies an event was linked to its time of occurrence and candidate description, if available.

### Medical event tagging

Given the cross-domain medical dictionary constructed in the previous section, we deployed a simple recursive algorithm that performs the longest match to recognize terms in each sentence. For a given sentence, we considered all possible phrases in this sentence, from the longest to the shortest, and used a Boolean array to mark the tagged characters. When tagging a sequence, we first check whether the first and last character of this sequence has been tagged. If either of them has been tagged, there is no need to tag it. This is because there’s a longer sequence that contains all or part of this sequence. Otherwise, we check whether one of the medical term lexica contains this sequence. If so, the sequence is added to the tagged list.

### Algorithm 1: Term Tagging

**Input**:

K, the set of 5 lexica

s, the sequence we are going to label

**Output**:

L, the list of tagged terms

1. **function** tag (s)

2.  n ← length of s;

3.  **declare** a Boolean array of length n has_tagged and set all values to false.

4.  **declare** an empty list L;

5.  **for** i = n to 1

6.   **for** j = 1 to n - i + 1

7.    **if** has_tagged[j] = false and has_tagged[j + i - 1] = false **then**

8.     s_0_ ← substring of s that starts from j and spans i characters;

9.     **foreach** dictionary D ∈ K

10.      **if** s_0_ ∈ D **then**

11.       add {s_0_, D} to L;

12.       **for** t = j to j + i– 1

13.        has_tagged[t] ← true;

14.  **return** L

An EMR is used to describe a single patient's medical history. Therefore, we assume the medical terms that appear in the EMRs describe medical events the patient has experienced with a few exceptions, namely symptoms, procedures and clinical tests. For example, “无恶寒” (no chills) appears in Fig 1a. Although “chills” is a symptom, it is not a medical event in this context. “拟行前列腺电切术” (Scheduled for transurethral resection of the prostate) means that the patient hasn’t had this operation yet. Thus, we deployed a rule-based filter to remove medical terms prefixed with negations including “no”, “refuse to”, “deny” and “haven’t seen”, or words and phrases indicating future actions including “scheduled to” and “suggest to”.

### Temporal term extraction

Temporal terms were used to describe medical events. A temporal term may consist of up to nine parts:

- Prepositional approximation words (Pre-AW) such as “约” (about),
- Postpositional approximation words (Post-AW) such as “左右”(around),
- Numbers,
- Unit such as “个”(piece),
- Temporal unit such as “年”(year),
- Prepositions such as “前”(before),
- A certain day such as “yesterday”,
- A certain period in a day such as “afternoon”, and
- A certain time in a day such as “15:00”.

Then temporal terms can be described by regular expressions as follows:

- (Pre-AW)? Numbers (Unit)? (Temporal unit) (Post-AW)? Preposition
- (A certain day) (A certain period in a day)? ((Pre-AW)? (A certain point in a day) (Post-AW)?)?

In addition, six Chinese idiomatic terms not covered by the regular expressions, “昨夜”(last night), “昨晚”(yesterday evening), “今夜”(tonight), “今晚”(this evening), “今早”(this morning) and “今晨”(this morning), were added.

The “coverage” of temporal terms can be summarized by the following scoping rules:

- If a statement contains a time point, the time covers the whole statement.
- If a statement contains multiple time points, each time point covers the segment before the next time point appears.
- If a statement contains no time points, the last time point appearing in the previous statement applies to the entire statement.

Here a statement referred to the segment of text up to a period symbol. All medical events in the scope covered by time *t* were assumed to have taken place at time *t*.

### Matching auxiliary descriptions

For a given EMR, which contains a temporal term and medical event, the remaining segment of terms constitutes a candidate description of the medical event. Furthermore, we constructed a set of rules to filter out the following sequences which do not constitute a Description:

- Sequences containing any medical term which is not considered to be a medical event, like “无恶寒”(no chills);
- Sequences containing some common words in EMRs, including “入院” (hospital admission), “出院” (discharge from hospital), “住院” (hospitalize), “就诊” (visit a doctor) and “治疗” (receive treatment);

Then, to associate a candidate description to the correct medical event, we generated all possible (time-event-description) triples and classified these triples as either true or false by a binary Support Vector Machine (SVM). One description may refer to multiple events and multiple descriptions may refer to the same event. This is the only supervised component of this framework that requires some human labeling.

In order to link the medical event and description, eight features were defined, consisting of local context features and semantic features:

1. Local context features:
  - Three words before and after each recognized term;
  - Three words before and after each candidate description.
  - Whether a candidate description contains digits;
2. Semantic features:
  - Normalized Google Distance [39] between a medical term x and a candidate description y is
    $$\text{NGD(x,y)}=\frac{\text{max}\{\text{log}f(x),\text{log}f(y)\}-\text{log}f(x,y)}{\text{log}M-\text{min}\{\text{log}f(x),\text{log}f(y)\}},$$
    where M is the total number of webpages indexed by a search engine; f(x) and f(y) are the number of search results when searching for x and y individually, respectively; and f(x, y) is the number of webpages where both x and y occur;
  - Number of recognized terms between two terms;
  - Number of candidate descriptions between two terms;
  - Number of commas between two terms;
  - The type of recognized term.

Feature 1 and 2 in local context features and the last feature in semantic features were also used in Patrick and Li [27].

## Results and Discussion

We evaluated the proposed framework on a real world dataset of 24,817 Histories of Present Illness (HPI). We first demonstrated the performance of each individual step, namely the recognition of medical terms by the core lexica, the enriching of the lexica by data-driven methods, the temporal relationship recognition and description matching process using SVM. We then present the results of the end-to-end extraction task.

### Core lexica and concept recognition

The six core lexica contain 31493 disease terms, 3723 symptom terms, 36725 drug terms, 6666 body part terms, 5758 procedure terms and 1019 clinical test terms. The result of term recognition using the core lexica is shown in Table 1. An F1-score is satisfactory in symptom and clinical test categories but relatively low in the disease, drug, body part, and procedure categories.

**Table 1:** Term recognition by the core lexica.

	Number of terms in GD-400	Number of recognized terms	Number of correctly recognized terms	Recall (%)	Precision (%)	F1-score (%)
Disease	1179	1069	810	68.8	75.7	72.1
Drug	858	774	707	82.4	91.3	86.6
Body Part	752	1678	556	73.9	33.1	45.8
Procedure	117	100	60	51.3	60.0	55.3
Symptom	4970	4919	4395	88.4	89.3	88.9
Clinical Test	1143	1172	1042	88.9	91.2	90.0
Total	9019	9712	7570	83.9	77.9	80.8

### Lexicon enrichment

First, we demonstrate the effect of pattern iteration updates on disease lexica and drug lexica. In the Chinese EMR, the words around a disease or a drug term are usually useful for determining whether the term is a drug or disease, such as 诊断为II型糖尿病 (diagnosed with type II diabetes) or 予舒弗美口服 (prescribe theophylline to take orally). The other four categories do not have this type of distinguishable pattern. For example, clinical tests often appear in this way “胸片:” (Chest X-ray:). In the Chinese EMR, a colon cannot provide sufficient evidence to determine whether the word before it is a clinical test term. The text after the colon also varies significantly depending on the type of clinical tests. Therefore, pattern iteration was used to enrich only the disease and drug lexica.

**Fig 5:**
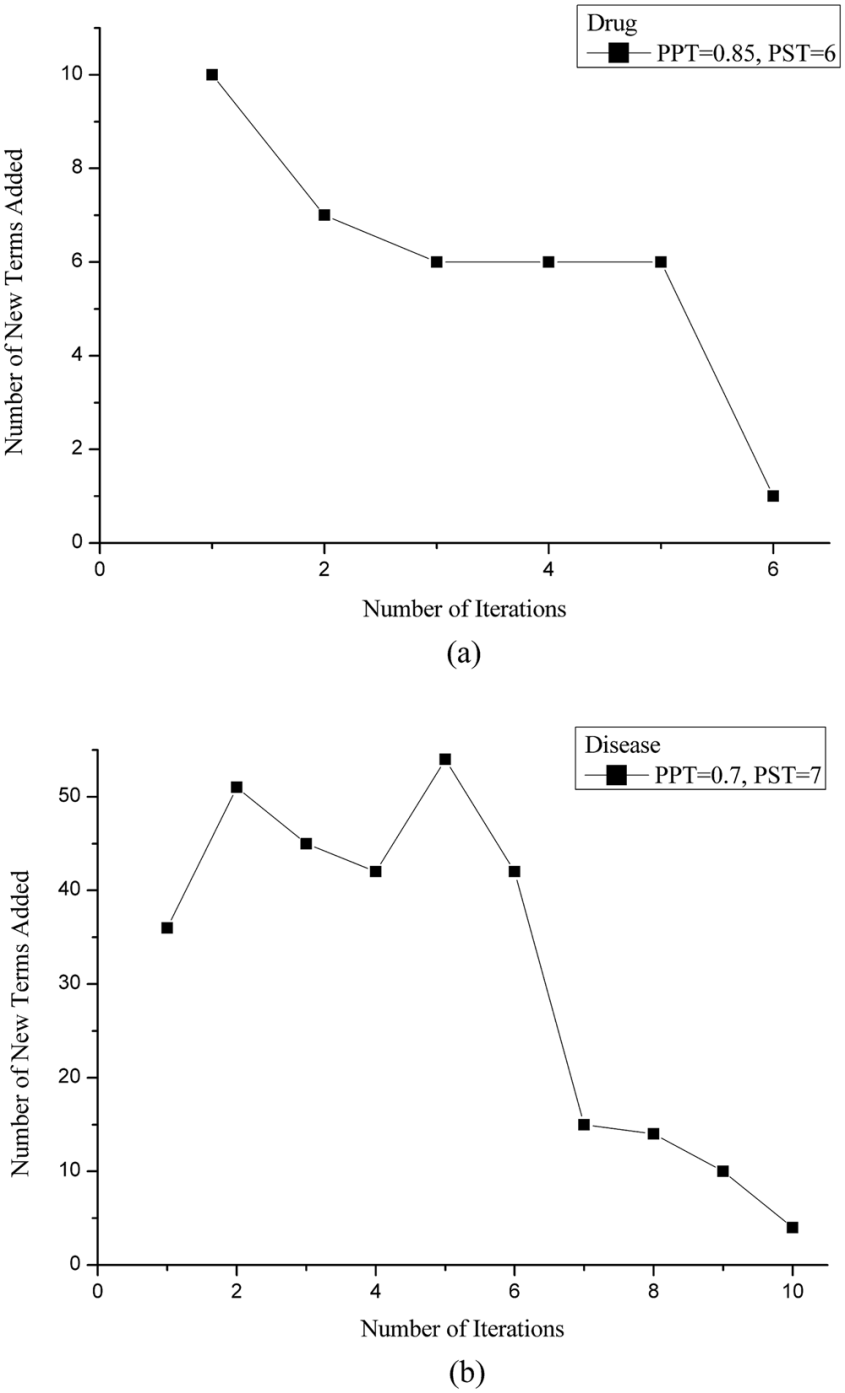
Number of new terms added with each iteration. Number of new (a) drug and (b) disease terms added in each iteration with the fixed threshold that yields the best F1-score.

Fig 5 shows the effect of pattern iteration on the disease and drug lexica. “PST = 7, PPT = 0.7” and “PST = 6, PPT = 0.85” were chosen via grid-search for disease and drug lexica, respectively, as they optimized the F1-score. Fig 5a displays the number of new disease terms added, respectively, with each iteration. Fig 5b shows the number of new drug terms added in each iteration. We can see that the algorithm converged in no more than 10 iterations. The effect of pattern iteration on term recognition can be found in Table 2. The recall in both the disease and drug lexica improved significantly. Lexica whose F1-scores have improved are highlighted. We can also find that pattern iteration has a slight impact on body part and symptom recognition. The reasons are as follows. Some diseases start with a body part, so body part recognition is slightly affected when more diseases are recognized. On the other hand, the iteration step may introduce erroneous terms, some of which contain symptoms in the core lexica, so the F1-score became slightly lower than that in the core lexica.

**Table 2:** Term recognition after pattern iteration.

	Recall (%)	Precision (%)	F1-score (%)
Disease	79.4	76.2	**77.8**
Drug	87.5	91.9	**89.8**
Body Part	71.0	34.2	**46.1**
Procedure	51.3	60.0	55.3
Symptom	87.5	89.5	88.5
Clinical Test	88.9	91.2	90.0
Total	84.8	79.0	81.8

Table 3 shows the improvement in term recognition after applying the body part prefix. In addition to having a positive effect on the body part lexicon, this technique also improves the results of term recognition in the disease, symptom, procedure and clinical test categories. In Chinese, it is very common for these four category terms to start with a body part. It doesn’t have any impact on drug terms, as compound drug terms usually do not contain body part prefixes.

**Table 3:** Term recognition after prefix enrichment.

		Recall (%)	Precision (%)	F1-score (%)
Disease	After body part prefix	80.0	768	78.4
	After directional or extensional prefix	85.6	822	**83.9**
Drug	After body part prefix	87.5	91.9	89.8
	After directional or extensional prefix	87.5	91.9	89.8
Body Part	After body part prefix	64.7	47.3	54.1
	After directional or extensional prefix	79.0	57.1	66.3
Procedure	After body part prefix	53.8	63.6	58.3
	After directional or extensional prefix	54.7	65.3	59.5
Symptom	After body part prefix	90.3	92.4	91.4
	After directional or extensional prefix	93.3	95.5	**94.4**
Clinical Test	After body part prefix	96.0	93.6	94.8
	After directional or extensional prefix	96.7	94.3	95.5
Total	After body part prefix	86.8	85.1	85.9
	After directional or extensional prefix	90.5	89.6	90.0

Term recognition results after a directional or extensional prefix update can be found in Table 3. This technique makes significant improvements on disease and symptom terms, for directional or extensional prefixes are very common in Chinese symptom and disease terms, such as “右下腹疼痛” (literally “right low abdominal ache”), “双肺感染” (literally “double lung infection”).

The resulting enriched lexica contains 33803 disease terms, 36792 drug terms, 9551 body part terms, 6092 procedure terms, 6306 symptom terms and 1386 clinical test terms.

Table 4 shows the recall/precision/F1-score of term recognition of the enriched lexica compared with the CRF method by Lei *et al* [24], the state-of-art medical event recognition method in Chinese EMRs. Lei *et al* provides two sets of evaluation measures—exact match and inexact match. Exact match means that an entity is considered correctly predicted if and only if the starting and ending offsets are exactly the same. While the inexact measure means that an entity is considered correctly predicted if it overlaps with any entity in the GD-400. We can see that the enriched lexica worked better in 5 of the 6 domains compared with the exact match measurement and slightly less in the “Body Part” domain. Even compared with the inexact match measurement, the enriched lexica achieved a 1% improvement in the “Drug”, “Symptom” and “Clinical Test” domains. In general, the enriched lexica achieved better recall than the CRF while the CRF did better with precision.

**Table 4:** Comparison of two methods in term recognition: recall, precision and F1-score.

	Our method (enriched lexica)	Lei *et al* (CRF) Inexact-match	Lei *et al* (CRF) Exact-match
Disease	83.9 (85.6/82.2)	84.8 (80.6/89.6)	80.2 (76.2/84.7)
Drug	89.8 (87.5/91.9)	88.2 (80.2/98.2)	85.5 (77.6/95.1)
Body Part	66.3 (79.0/57.1)	68.4 (59.1/81.2)	66.9 (57.8/79.4)
Procedure	59.5 (54.7/65.3)	60.0 (47.1/82.6)	55.7 (43.8/76.8)
Symptom	94.4 (93.3/95.5)	94.0 (92.4/95.6)	93.4 (91.9/95.1)
Clinical Test	95.5 (96.7/94.3)	94.7 (92.3/97.2)	94.2 (91.8/96.8)
**Total**	89.6 (90.5/88.6)	89.9 (86.1/94.2)	88.6 (84.7/92.8)

Values are F1-score (recall/precision) (%)

In terms of F1-scores, our method is significantly better than Lei’s CRF, while comparing to the exact match measurement, with an average difference in F1-score of 0.0225 among the 6 categories (one-sided 95%CI: 0.0063–∞, p-value = 0.0189).

### Temporal relationships

We have detected 1338 temporal terms in GD-400. 98.5% of the medical events were linked to the correct times. The recall and precision of temporal term recognition were 0.967 and 1.0, respectively. Temporal terms that were not recognized correctly were made up of period terms that had been confused as fixed time points.

### Auxiliary descriptions

The NGD matrix was constructed using the Microsoft Bing search engine, due to the inaccessibility of Google in China. The performance was assessed using 10-fold cross validation. Here we only evaluated the triples that the SVM predicted as true, as we were exploring an extraction problem where false predictions are not meaningful. Results showed a recall of 0.892, a precision of 0.856 and an F1-score of 0.874.

As a comparison, we designed a simple baseline which associates the medical event with the candidate description immediately after that medical event. Table 5 shows that the F1-score of the baseline is 0.761, which is less than 0.113 compared to the NGD SVM. Also, we implemented the SVM model by Patrick and Li using four local context features and two semantic features [23], which is the most recent study on entity linking in EMRs to the best of our knowledge. Additionally, we compared the NGD SVM with orwithout NGD feature. The results are shown in Table 5. We found that the NGD feature achieved a 3.6% improvement in F1-score, which is significant. And compared with Patrick *et al*, the NGD SVM was superior in terms of precision (0.856 vs. 0.788) and comparable in terms of recall (0.892 vs. 0.895) and also had a much improved F1-score (0.874 vs. 0.838).

**Table 5:** Detailed results of auxiliary description matching on GD-400.

Method	Recall (%)	Precision (%)	F1-score (%)
Baseline	71.2	81.6	76.1
**NGD SVM**	89.2	85.6	**87.4**
SVM without NGD feature	86.8	80.9	83.8
Patrick and Li	89.5	78.8	83.8

### End-to-End result

The end-to-end performance of the framework was assessed on GD-400. The output of the framework is provided in the time-event-description triples as shown in Fig 1b. We divided GD-400 into 4 parts, 100 records each. Then, we used 300 records to train the SVM and the others for testing. We extracted 6476 records and 5528 of them were correct. The end-to-end result showed a recall of 0.838, a precision of 0.854 and an overall F1-score of 0.846. Furthermore, we matched each medical event with its time of occurrence and description, where applicable.

### Data-driven approach to address Chinese and medical writing conventions

The proposed approach is largely data-driven. For a given set of Chinese EMRs, the approach is capable of automatically building a medical lexicon from an EMR corpus on the basis of static core lexica. It automatically generates high quality patterns to discover new terms in a large body of EMR text and simultaneously mines new patterns iteratively. However, if low quality patterns are added, the accuracy of newly discovered terms will be affected. The lexica are further improved through an automatic discovery mechanism, which uses body part prefixes and directional or extensional prefixes in both the EMR corpus and the existing term lexica. The advantage of the lexicon enrichment process is two-fold. First, Chinese medical text writing conventions and variations in writing styles are captured through patterns used for discovering new terms and rules for updating the lexica. Second, no human annotation of the text is required in the lexicon enrichment process, which makes the approach scalable to handle large quantities of EMRs.

### Temporal and auxiliary descriptions of medical events

In the proposed framework, temporal and auxiliary descriptions of medical events were captured using rules and machine learning based algorithms. These descriptions for medical treatments are often hard to capture. This is because: i) they occur at lower frequencies than other terms and; ii) they are difficult to cover by lexica. However, they can be important to downstream analysis. For instance, a “dawn phenomenon” is an early morning increase in blood glucose in patients with diabetes, which is not associated with nocturnal hypoglycemia. To define a dawn phenomenon, it is important to determine when hyperglycemia was observed and when there was no nocturnal hypoglycemia in the evening before a significant increase of blood sugar. A patient who coughs before sleep in bed might have left ventricular heart failure, while a patient who coughs during the morning is more likely to have chronic pulmonary obstructive disease. Furthermore, the word “badly” in “coughing badly” might suggest the severity of a respiratory infection. One limitation in this part of the work is that we have a relatively simple rule-based model to detect medical events, where a more advanced method may be attempted in future.

### Using non-domain-specific NGD

The innovative feature incorporated into our methodology is the use of Normalized Google Distance, which measures the approximate degree of relatedness of any two terms by leveraging large amounts of indexed documents with search engines. When we compute event-to-description correlation, the descriptions can be very diverse, sometimes not exactly medical terms or even colloquial. Existing medical texts or EMR corpus often do not have good enough coverage to include all those descriptions. Whereas a commercial search engine like Google or Bing indexes large enough amounts of data to overcome this problem. Despite the relatively high cost of communication to obtain NGD by querying search engines, this process can be done offline, and most importantly, such a “big data” approach significantly improves the accuracy of the description-matching task.

## Conclusion

We have introduced a delicate framework for extracting structured records from free text narratives in Chinese EMRs. The framework employs a data-driven principle while integrating rule-based techniques and machine learning algorithms. We obtained an F1-score of 0.846 on end-to-end extraction of time-event-description triples from 400 randomly selected records out of a dataset of 24,817 EMRs.

The data-driven and overall weakly-supervised nature of the framework makes it suitable for large EMR datasets. It addresses the Chinese medical writing convention and variations in writing medical narratives through data-driven and rule-based updates to the core lexica. When applicable, each medical event is matched to a time point and an auxiliary description, which may contain information essential for downstream analyses. Including NGD in the SVM algorithm for description matching is another unique strength of the framework. The results of our experiments have shown that, despite coming from general webpages, NGD has proven to be a useful source of information for event domain specific information extraction.
